# Hematological Parameters and Hemozoin-Containing Leukocytes and Their Association with Disease Severity among Malaria Infected Children: A Cross-Sectional Study at Pawe General Hospital, Northwest Ethiopia

**DOI:** 10.1155/2017/8965729

**Published:** 2017-02-19

**Authors:** Muluken Birhanu, Yaregal Asres, Wondimagegn Adissu, Tilahun Yemane, Endalew Zemene, Lealem Gedefaw

**Affiliations:** ^1^Department of Medical Laboratory Science, Assosa University, Assosa, Ethiopia; ^2^Department of Medical Laboratory Science and Pathology, College of Health Sciences, Jimma University, Jimma, Ethiopia

## Abstract

Hematological parameter changes are the most common complications in malaria. We aimed to determine the hematological parameters and hemozoin-containing leukocytes and their association with disease severity in malaria infected children aged between 1 and 15 years. A facility-based cross-sectional study was conducted at Pawe General Hospital from July 31 to December 30, 2014. Demographic and clinical data were collected using structured questionnaire. Blood specimen was collected from each study participant for hematological investigations. Data were analyzed using SPSS version 20. The overall prevalence of anemia was 40.3%, most of which were mildly anemic. Leukocytosis was found in 15.4% of study participants. More than a fourth (27%) of the children had severe malaria. Hemozoin-containing monocytes and neutrophils were found in 80.1% and 58.9% of the study participants, respectively. Under-five years of age (AOR = 3.01, 95% CI: 1.83–7.39, *P* < 0.001), leukocytosis (AOR = 3.20, 95% CI: 1.65–6.24, *P* = 0.001), mean hemozoin-containing monocytes >5% (AOR = 6.26, 95% CI: 2.14–14.29, *P* < 0.001), mean hemozoin-containing neutrophils >5% (AOR = 7.93, 95% CI: 3.09–16.86, *P* < 0.001), and high density parasitemia (AOR = 1.90, 95% CI: 1.13–3.18, *P* = 0.015) were associated with severe malaria. Hemozoin-containing leukocytes, leukocytosis, and other identified associated factors should be considered for proper management of children with severe malaria.

## 1. Introduction

Malaria is one of the infectious diseases that may affect hematological parameters. Anemia, thrombocytopenia, leukocytosis, and leukopenia are the most common hematological complications associated with malaria [1–3]. The extent of these alterations varies with malaria endemicity, background hemoglobinopathy, nutritional status, demographic factors, and malaria immunity [3–5]. Hemozoin (HZ) is the end product of hemoglobin (Hb) digestion by* Plasmodia*. Both circulating and resident phagocytes acquire HZ through phagocytosis of parasitized red blood cells (PRBCs). Free HZ crystals may also be released after schizont rupture. Some studies show acquisition of HZ by circulating monocytes and neutrophils to be associated with malarial severity [6, 7]. Detection of HZ pigment within these cells may help in the diagnosis of malaria and is considered as one of the signs of its severity [8–11].

Hematological changes are the most common complications in malaria and play major role in disease outcomes. In malaria endemic areas, severe malarial anemia (SMA) is one of the major factors contributing to anemia resulting in morbidity and mortality among children [4, 5]. Health facility-based data show that up to 15% total deaths among children under five years of age in sub-Saharan Africa (SSA) is attributable to anemia [12]. Malarial anemia is also a major public health problem in Ethiopia, with 43.4% of under-five children with malaria being anemic [13]. Health facility-based studies reported SMA ranging from 13.3% to 37.8% among children with SM [14–16]. Moreover, prevalence of mean HZ-containing monocytes (HCMs) and HZ-containing neutrophils (HCNs) ranging from 4% to 53% and 2% to 27% have been reported among children with SM in Africa, respectively [7, 17–19].

Although there are several factors that increase mortality due to malaria in Ethiopia, the absence of reliable diagnosis and management of SM is the most important. In health facilities in Ethiopia, SM is usually diagnosed by counting the number of asexual parasitaemia in peripheral blood smear of the patients [15]. However, peripheral parasitemia alone is a poor indicator of disease severity, and in many cases the severity of MA does not directly correlate with the number of circulating parasites [20], owing to sequestration within the microvasculature of the host. Some studies reported association of HCMs [10], thrombocytopenia, and leukocytosis [21–23] with SM. On the other hand, no association of platelet (PLT) and white blood cell (WBC) counts with malaria severity was found in other studies [24, 25]. The presence and extent of hematological abnormalities in children with malaria and their association with disease severity were not well studied in Ethiopia. Hence, this study was aimed at determining the hematological parameters and HZ-containing leukocytes (HCLs) in children with malaria and their association with disease severity at Pawe General Hospital.

## 2. Materials and Methods

### 2.1. Study Design and Setting

A facility-based cross-sectional study was conducted at Pawe General Hospital from July 31 to December 30, 2014. Pawe General Hospital is found in Metekel Zone of Benishangul Gumuz Regional State, located 557 kms northwest of Addis Ababa. The hospital serves an estimated 300,000 people in the region, with bed capacity of 180. The geographical coordinates of the area are approximately 11°19′N latitude and 36°25′E longitude, with altitude range of 1050–1250 meters above sea level. The annual mean rainfall is 1150 mm with average temperature of 32°C. The area is characterized by hot and humid climatic condition with stable, year-round transmission of malaria. Even though there is perennial transmission of malaria in the study area, the transmission peaks from September to December, following the main rainy season from June to September [13, 26].

### 2.2. Study Population and Sampling

A total of 377 children diagnosed with malaria by peripheral smear microscopy at the hospital were included in this study. The sample size was estimated using single population proportion formula considering 43% expected prevalence of malarial anemia [14], 5% marginal error, and 95% confidence level. Children of both sexes aged between 1 and 15 years with microscopy-confirmed malaria diagnosed at the outpatient department of the hospital during the study period were included consecutively. Written consent was obtained from parents/guardians before enrollment in the study.

The following exclusion criteria were considered in this study: (1) positivity for intestinal parasite(s) as screened by microscopic examination of wet mount and formol-ether concentrated stool sample; (2) HIV-1/2-seropositivity as screened by the HIV rapid diagnostic tests (KHB, STAT-PAK, and Uni-Gold); (3) antimalarial treatment 48 hours prior to data collection; (4) history of chronic illness as responded by the parents/guardians of the children.

### 2.3. Data Collection

Demographic and clinical data of the children were collected by a trained public health officer using structured questionnaire. Moreover, laboratory data including complete blood count (CBC), blood film examination for hemoparasites, and HCLs were generated in this study. Approximately 4 mL of venous blood was aseptically collected into ethylene diamine tetra acetic acid- (EDTA-) containing vacationer tube. The blood samples were analyzed for CBC and thin and thick blood smears prepared for microscopic examination of malaria parasites. The CBC was analyzed using CELLDYNE 1800® (Abott Laboratories Diagnostics Division, USA) within two hours of sample collection. Thick and thin blood films were prepared on single slide for each study participant. Thin blood smears were methanol-fixed before staining. The slides were stained using 10% Giemsa stain (PH 7.2) for 10 minutes, with the Giemsa stain being prepared immediately before use. The blood smears were examined for malaria parasites, and HCLs were enumerated.

To ensure quality of the data generated in the study, data collectors were trained prior to commencement of the study. Control samples were utilized to ensure accuracy of outputs of the hematology analyzer. Two independent blinded laboratory technologists examined the blood films. In case of discrepancy between the two readings, a third person was involved in reading the slide. Moreover, 10% of the slides were randomly selected and reexamined by the third laboratory technologist.

### 2.4. Data Processing and Analysis

Children with at least one of the clinical symptoms: prostration, severe malarial anemia, and hyperparasitemia were classified to have SM [27]. Malaria parasite density was graded as low, moderate, and high, when the parasite count was between 1–999/*μ*L, 1,000–10,000/*μ*L, and >10,000/*μ*L, respectively [28]. Children with Hb concentration of 5–11 g/dL and <5 g/dL were considered to have malarial anemia and severe malarial anemia, respectively [29]. Leukopenia and leukocytosis were considered when the total WBC count was <4.0 × 10^9^/L and >11.0 × 10^9^/L, respectively. Thrombocytopenia was defined as PLT count <150 × 10^9^/L [30].

The collected data were checked for completeness regularly during the course of data collection. The data were entered, cleaned, and analyzed using SPSS version 20.0 (SPSS Inc., Chicago, IL). Descriptive statistics including frequency, mean, and standard deviation were utilized to summarize demographic profile of the children and some of the clinical data. Bivariate and multivariable logistic regressions were used to assess association of the independent variables with the dependent variables. Variables with *P* value less than 0.25 were candidates for the multivariable logistic regression in this study. *P* value < 0.05 was considered significant during the analysis.

### 2.5. Ethical Considerations

Ethical clearance was obtained from Jimma University Ethical Review Board. Permission was obtained from the Regional Health Bureau. Voluntary participation of the children and their parents/guardians was ensured. Written informed consent was obtained from the parents/guardians of the children before enrollment in the study. Confidentiality of all the clinical information obtained from the children was strictly maintained. All the children who were involved in the study were treated for malaria according to the Malaria Treatment Guidelines [31]. Moreover, children who had intestinal parasitic infection(s) were treated at the hospital.

## 3. Results

### 3.1. Demographic and Clinical Profiles of the Study Participants

A total of 377 malaria infected children aged 1 to 15 years were included in this study. The mean age of the study participants was 8.5 ± 4.9 years. A third of the study participants (33.7%, *n* = 127) were children between 1 to 5 years. At presentation, 63.9% (*n* = 241) of the children had fever (axillary temperature ≥ 37.5°C). The majority of malaria cases were due to* P. falciparum* (75.1%, *n* = 283) (Table 1).

### 3.2. Hematological Parameters and Hemozoin-Containing Leukocytes

Mean values of the hematological parameters are presented in Table 2. Hemozoin-containing monocytes and HCNs were found in 80.1% (*n* = 302) and 58.9% (*n* = 222) of study participants, respectively. On average, 9.7 and 1.7% of the monocytes and neutrophils contained HZ, respectively. The total HCMs and HCNs per microliter of blood of the study participants were 80.6 ± 34.3 and 85.8 ± 37.1, respectively. Children with SM had significantly lower mean Hb concentration, hematocrit value, and RBC count. On the other hand, the children with SM had significantly higher WBC count, mean HCMs count, and HCNs percentages compared to those with uncomplicated malaria. The mean PLT count did not differ significantly between the two groups (Table 2).

### 3.3. Factors Associated with Severe Malaria

Factors associated with severe malaria among the children are demonstrated in Table 3. Out of the total study participants, 40.3% (*n* = 152) were anemic, of whom 87.5% (*n* = 133), 9.2% (*n* = 14), and 3.3% (*n* = 5) had mild, moderate, and severe anemia, respectively. Thrombocytopenia was found in 56.8% (*n* = 214) of the study participants. Mild, moderate, and severe thrombocytopenia accounted for 41.6% (*n* = 89), 45.3% (*n* = 97) and 13.1% (*n* = 28) of the children with thrombocytopenia, respectively. Leukocytosis and leukopenia were found in 15.4% (*n* = 58) and 10.3% (*n* = 39) of the study participants, respectively.

After adjusting for all the measured explanatory variables in the multivariable regression model, under-five years of age (AOR = 3.01, 95% CI: 1.83–7.39), leukocytosis (AOR = 3.20, 95% CI: 1.65–6.24), mean HCMs >5% (AOR = 6.26, 95% CI: 2.14–14.29), mean HCNs >5% (AOR = 7.93, 95% CI: 3.09–16.86), and high parasitemia (AOR = 1.90, 95% CI: 1.13–3.18) were associated with SM.

## 4. Discussion 

The main aim of this study was to determine the common hematological indices and HCLs among the malaria infected children. Accordingly, the overall prevalence of anemia was 40.3%. Anemia is a common complication associated with malaria [4, 14, 30, 32], specially associated with falciparum malaria. The magnitude of anemia among malaria infected patients varies depending on demographic factors, nutritional status, preexisting physiologic conditions, and coinfection with other diseases [33, 34]. It is revealed in this study that the mean Hb concentration was significantly lower in children with SM compared to children with UM. The causes and mechanisms of anemia in malaria are diverse, which may include RBC destruction and effect on erythroid development [35, 36]. The high prevalence of anemia among the children requires urgent attention to curb its long-term impacts.

In this study, nearly half of the children had moderate to mild thrombocytopenia. Low PLT associated with malaria in malaria endemic areas has also been reported previously [4, 37–41]. The exact mechanism of pathogenesis of malarial thrombocytopenia is not clear. Suggested mechanisms of thrombocytopenia in malaria include splenic phagocytosis of activated platelets, coagulation disturbances, and bone marrow alterations [42, 43]. Unless treated early, thrombocytopenia may also play role in the pathogenesis of cerebral malaria [3].

Leukocytosis and leukopenia were found in 15.4% and 10.3% of the study participants, respectively. Moreover, children who had leukocytosis were 3 times more likely to have SM. Other studies also show significant association of leukocytosis with SM [22, 23, 37]. Leukocytes, particularly the monocytes and to a lesser extent neutrophils, play crucial role in the host defense against malaria parasites. As the severity of the disease progress, there might be overstimulation of the leukocytes resulting in imbalanced and excessive production of inflammatory cytokines, which may further contribute to the severity of the disease [44, 45].

In this study, it was also revealed that most of the malaria infected children had HCMs and HCNs in their peripheral blood smear. The interaction of HZ with immune cells may result in upregulation or downregulation of immune mediators [46–48] affecting host innate and inflammatory immune responses. Hemozoin may also be involved in the inhibition of erythropoiesis in malarial anemia [49, 50], likely by stimulation of apoptosis of erythroid precursors [51]. The high proportion of children with HCLs in this study requires vigilance over the course of the disease in this area, as HCLs could be associated with SM [9]. Evaluation of duration of persistence of HZ within the mononuclear cells and polymorphs following parasite clearance was not within the scope of this study. A previous study reported persistence of HZ in spleen and liver in mouse models, after parasite clearance [52].

The findings of this study should be interpreted in light of the following limitations. As no locally established reference range for hematological parameters in children was obtained, the WHO reference range was utilized. Although certain bacterial infections can profoundly perturb hematological parameters, thereby affecting their diagnostic value in determining severity of malaria, this study did not investigate any bacterial coinfections.

## 5. Conclusion

Over a quarter of the children with malaria had SM. Demographic and hematological parameters significantly affected the malaria disease outcome among the studied children. The presence and quantities of HCLs and other hematological parameters should be considered in the management of children with malaria in the study area. Further longitudinal studies with larger sample size are needed to determine the association of hematological parameters with SMA.

## Figures and Tables

**Table 1:** Demographic and clinical characteristics of the study participants, Pawe General Hospital, 2014.

Variables	Categories	Frequency	Percent (%)
Age (years)	<5	127	33.7
	≥5	250	66.3

Sex	Male	218	57.8
	Female	159	42.2

Axillary temperature	<37.5°C	151	40.1
	≥37.5°C	226	59.9

*Plasmodium* species	*P. falciparum*	283	75.1
	*P. vivax*	57	15.1
	Mixed^*∗*^	37	9.8

Malaria severity	Uncomplicated malaria	275	72.9
	Severe malaria	102	27.1

^*∗*^Mixed refers to *P. falciparum* and *P. vivax* coinfection.

**Table 2:** Comparison of mean values of hematological parameters and hemozoin-containing leukocytes with malaria severity among the study participants, Pawe General Hospital, 2014.

Parameters	Total mean (SD)	UM	SM	*T*-value^*∗*^	*P* value
Hb concentration (g/dL), *n* = 377	11.7 (2.2)	12.1 (1.9)	10.6 (2.4)	6.310	<0.001
Hct (%), *n* = 377	32.9 (5.6)	34.02 (4.8)	30.05 (6.6)	5.538	<0.001
RBC count (×10^12^/L), *n* = 377	4.4 (0.7)	4.51 (0.6)	4.18 (0.9)	3.237	0.002
WBC count (×10^9^/L), *n* = 377	7.7 (3.7)	7.09 (3.3)	9.31 (4.3)	−5.436	<0.001
PLT count (×10^9^/L), *n* = 377	153.0 (91.4)	151 (80.0)	160 (116)	−0.735	0.464
HCMs (%), *n* = 302	9.7 (9.2)	7.3 (7.2)	16.2 (10.9)	−7.618	<0.001
HCNs (%), *n* = 222	1.7 (2.2)	1.25 (1.6)	2.8 (3.1)	−4.685	<0.001
HCMs count (per *μ*L), *n* = 302	80.6 (34.3)	48.8 (46.1)	166.3 (82.1)	−5.327	<0.001
HCNs count (per *μ*L), *n* = 222	85.8 (37.1)	56.2 (69.8)	165.6 (80.5)	−5.140	<0.001

Hb = hemoglobin, Hct = hematocrit, HCMs = hemozoin-containing monocytes, HCNs = hemozoin-containing neutrophils, PLT = platelet, RBC = red blood cell, SD = standard deviation, SM = severe malaria, UM = uncomplicated malaria, WBC = white blood cell, and *μ*L = microlitter.

*∗* indicates Student's *t*-test.

**Table 3:** Factors associated with of severe malaria among the study participants, Pawe General Hospital, 2014.

Variables	Categories	UM: *N* (%)	SM: *N* (%)	COR (95% CI)	*P* value	AOR (95% CI)	*P* value
Age (year)	<5	70 (55.1)	57 (44.9)	3.71 (2.30–5.97)	<0.001	3.01 (1.83–7.39)	<0.001
	≥5	205 (82.0)	45 (18.0)	1 (ref)		1 (ref)	

Sex	Male	160 (73.4)	58 (26.6)	1.05 (0.67–1.67)	0.818		
	Female	115 (72.3)	44 (27.7)	1 (ref)			

Axillary temperature (°c)	<37.5	113 (74.8)	38 (25.2)	1 (ref)			
	37.5–39.4	162 (71.7)	64 (28.3)	1.24 (0.76–2.04)	0.235	1.03 (0.57–1.84)	0.960

Anemia	Yes	93 (61.2)	59 (38.8)	2.68 (1.69–4.28)	<0.001	1.65 (0.93–2.92)	0.089
	No	182 (80.9)	43 (19.1)	1 (ref)		1 (ref)	

Leukocytosis	Yes	32 (55.2)	26 (44.8)	2.6 (1.46–4.63)	<0.001	3.20 (1.65–6.24)	0.001
	No	243 (76.2)	76 (23.8)	1 (ref)		1 (ref)	

Leukopenia	Yes	33 (84.6)	6 (15.4)	0.46 (0.19–1.13)	0.090	0.68 (0.25–1.88)	0.462
	No	242 (71.6)	96 (28.4)	1 (ref)		1 (ref)	

Thrombocytopenia	Yes	154 (72.0)	60 (28.0)	1.12 (0.71–1.78)	0.623		
	No	121 (74.2)	42 (25.8)	1 (ref)			

HCMs	No HCMs	70 (93.3)	5 (6.7)	1 (ref)		1 (ref)	
	0.5–5%	143 (77.7)	41 (22.3)	2.04 (0.63–6.57)	0.234	3.77 (1.39–10.20)	0.009
	>5%	62 (52.5)	56 (47.5)	8.31 (3.23–1.36)	<0.001	6.26 (2.14–14.29)	<0.001

HCNs	No HCNs	125 (80.6)	30 (29.4)	1 (ref)		1 (ref)	
	0.5–5%	143 (73.7)	51 (26.3)	1.49 (0.89–2.48)	0.129	0.79 (0.43–1.46)	0.455
	>5%	7 (25)	21 (75)	12.5 (4.86–2.12)	<0.001	7.93 (3.09–16.86)	<0.001

*Plasmodium* species	*P. falciparum*	191 (67.5)	92 (32.5)	5.56 (1.63–11.12)	0.006		
	*P. vivax*	50 (87.7)	7 (12.3)	1.59 (0.38–6.57)	0.524		
	Mixed^*∗*^	34 (91.9)	3 (8.1)	1 (ref)			

High parasitemia	Yes	117 (65.0)	63 (35.0)	2.18 (1.37–3.47)	0.001	1.90 (1.13–3.18)	0.015
	No	158 (80.2)	39 (19.8)	1 (ref)		1 (ref)	

UM = uncomplicated malaria, SM = severe malaria, *N* = number, COR = crude odds ratio, AOR = adjusted odds ratio, CI = confidence interval, HCMs = hemozoin containing monocytes, HCNs = hemozoin containing neutrophils, ref = reference, and *μ*L = microliter. ^*∗*^Mixed refers to *P. falciparum* and *P. vivax* coinfection.
